# Oxytocin receptor gene variations and socio-emotional effects of MDMA: A pooled analysis of controlled studies in healthy subjects

**DOI:** 10.1371/journal.pone.0199384

**Published:** 2018-06-18

**Authors:** Patrick Vizeli, Matthias E. Liechti

**Affiliations:** Division of Clinical Pharmacology and Toxicology, Department of Biomedicine and Department of Clinical Research, University Hospital Basel and University of Basel, Basel, Switzerland; Technion Israel Institute of Technology, ISRAEL

## Abstract

Methylenedioxymethamphetamine (MDMA) increases oxytocin, empathy, and prosociality. Oxytocin plays a critical role in emotion processing and social behavior and has been shown to mediate the prosocial effects of MDMA in animals. Genetic variants, such as single-nucleotide polymorphisms (SNPs), of the oxytocin receptor (OXTR) may influence the emotional and social effects of MDMA in humans. The effects of common genetic variants of the OXTR (*rs53576*, *rs1042778*, and *rs2254298* SNPs) on the emotional, empathogenic, and prosocial effects of MDMA were characterized in up to 132 healthy subjects in a pooled analysis of eight double-blind, placebo-controlled studies. In a subset of 53 subjects, MDMA produced significantly greater feelings of trust in *rs1042778* TT genotypes compared with G allele carriers. The *rs53576* and *rs225498* SNPs did not moderate the subjective effects of MDMA in up to 132 subjects. None of the SNPs moderated MDMA-induced impairments in negative facial emotion recognition or enhancements in emotional empathy in the Multifaceted Empathy Test in 69 subjects. MDMA significantly increased plasma oxytocin concentrations. MDMA and oxytocin concentrations did not differ between OXTR gene variants. The present results provide preliminary evidence that OXTR gene variations may modulate aspects of the prosocial subjective effects of MDMA in humans. However, interpretation should be cautious due to the small sample size. Additionally, OXTR SNPs did not moderate the subjective overall effect of MDMA (any drug effect) or feelings of “closeness to others”.

**Trial registration:** ClinicalTrials.gov: http://www.clinicaltrials.gov, No: NCT00886886, NCT00990067, NCT01136278, NCT01270672, NCT01386177, NCT01465685, NCT01771874, and NCT01951508.

## Introduction

3,4-Methylenedioxymethamphetamine (MDMA; ecstasy) is recreationally used for its effects on empathic feelings and sociability [1, 2]. MDMA has also been shown to reduce the perception of negative emotions and enhance empathy [1, 3–6], effects that could potentially be useful in MDMA-assisted psychotherapy [7]. MDMA mainly causes the release of serotonin, norepinephrine, and dopamine [8, 9] but also increases oxytocin [1, 10–14]. Many similarities are seen in the effects of MDMA and oxytocin on emotion processing and social behavior. For example, MDMA improves the identification of positive facial emotions and impairs the recognition of negative facial emotions [1, 4, 12, 15]. Similar effects were observed after intranasal administration of oxytocin [16, 17]. Intranasal administration of oxytocin increased trust and productive communication [18, 19]. MDMA similarly increased feelings of trust, openness, and closeness to others [1, 3]. Oxytocin increased generosity, and MDMA increased prosocial economic behavior [1]. One study showed significant within-subject correlations between MDMA-induced changes in prosocial feelings and changes in plasma oxytocin concentrations [10] implicating oxytocin as a mediator of the prosocial effects of MDMA. However, many other studies failed to find associations between oxytocin concentrations and the subjective, emotional, empathic, or prosocial effects of MDMA across subjects [1, 4, 12, 20, 21]. Animal studies showed that MDMA-induced prosocial effects in rats and mice could be blocked by oxytocin receptor (OXTR) antagonists [13, 22] also supporting a role for oxytocin in the prosocial effects of MDMA in animals. However, whether oxytocin is indeed a mediator of the effects of MDMA in humans is unclear. Oxytocin receptor blockade in the human brain may be challenging because OXTR blockers, such as atosiban, do not cross the blood-brain barrier. Correlational analyses are also problematic because plasma oxytocin concentrations may not necessarily reflect oxytocin levels in the brain [23].

While MDMA induces positive mood effects and prosociality in most subjects, negative mood effects have also been reported [24–27]. Genetic variations could explain some of the interindividual differences in the response to MDMA. Specifically, several genetic variations of the OXTR that are caused by single-nucleotide polymorphisms (SNPs) are associated with human social behavior or traits [28, 29]. One approach to indirectly testing the role of oxytocin in the effects of MDMA is to evaluate the moderating role of different SNPs of the OXTR gene in the prosocial and empathogenic effects of MDMA. In one study on the role of OXTR gene variants in the effects of MDMA, MDMA increased sociability in carriers of the G allele of the OXTR *rs53576* SNP but not in individuals with the AA genotype [30]. Furthermore in studies without MDMA, the common GG variant of *rs53576* has been associated with greater empathy and trust, seeking emotional support in times of distress, and less stress reactivity [31]. In contrast, carriers of the A allele presented lower sociability and lower sensitivity to oxytocin-induced enhancements in emotion recognition [32]. In addition to the *rs53576* SNP, two of the most studied SNPs of the OXTR gene are *rs1042778* and *rs2254298*. These SNPs were more likely to show an association to prosociality or other effects of oxytocin than other SNPs studied. For example, among 15 SNPs, the *rs1042778* showed the most significant associations with prosociality [33]. Carriers of the G allele of the *rs1042778* SNP presented greater prosocial behavior in an economic exchange game also used in the present study [33] and more sensitive parenting [34]. The *rs2254298* SNP has been associated with autism, attachment behavior, and depression [35–37] and *rs2254298* and *rs53576* were the most informative among 27 OXTR SNPs in a small sample of 38 subjects to predict responses to oxytocin in autism [38].

In the present study, we exploratorily investigated whether the OXTR *rs53576*, *rs1042778*, and *rs2254298* SNPs influence the social subjective, emotional, empathic, and prosocial effects of MDMA. Additionally, we sought to replicate previous findings regarding the role of the OXTR *rs53576* SNP in the prosocial response to MDMA [30].

## Materials and methods

### Study design

This was a pooled analysis of eight Phase I double-blind, placebo-controlled, crossover studies in healthy subjects that used similar methods [8, 39–45]. These studies included a total of 136 healthy subjects and were conducted between April 2009 and December 2014. Seven studies each included 16 subjects (112 total subjects) who received 125 mg MDMA twice, once alone and once after pretreatment with a medication [8, 39–43, 45]. However, in the present analysis, only data from the MDMA-alone and placebo sessions were used. An additional study included 24 subjects who received 125 mg MDMA once without pretreatment [44]. In all of the studies, the washout periods between the single dose administrations were at least 7 days to exclude carry-over effects. The studies were all registered at ClinicalTrials.gov (NCT00886886, NCT00990067, NCT01136278, NCT01270672, NCT01386177, NCT01465685, NCT01771874, and NCT01951508). All of the studies were approved by the local ethics committee and Swiss Agency for Therapeutic Products (Swissmedic). The studies were conducted in accordance with the Declaration of Helsinki. Informed consent was obtained from all of the participants who were included in the studies. All of the subjects were paid for their participation. Pharmacokinetic and safety data from the same studies have been reported elsewhere [26, 46, 47].

### Subjects

A total of 136 healthy subjects of European descent, aged 18–44 years (mean ± SD = 24.8 ± 4 years), were recruited from the University of Basel campus and participated in the study. One genotyping sample was missing, and three participants did not give consent for genotyping, resulting in the analysis of data from 132 subjects (64 men, 68 women). The mean ± SD body weight was 68 ± 10 kg (range: 46–90 kg).

The detailed exclusion criteria were reported elsewhere [8, 39, 40, 42] and included a history of psychiatric disorders, physical illness, a lifetime history of using illicit drugs more than five times (with the exception of past cannabis use), illicit drug use within the last 2 months, and illicit drug use during the study, determined by urine tests before the test sessions.

### Study drug

(±)MDMA hydrochloride (Lipomed AG, Arlesheim, Switzerland) was administered orally in a single dose of 125 mg that was prepared as gelatin capsules (25 and 100 mg, Bichsel Laboratories, Interlaken, Switzerland). Similar amounts of MDMA are found in ecstasy pills and have been used in clinical studies in patients [7]. The doses were not adjusted for body weight or sex. The dose per body weight (mean ± SD) was 1.9 ± 0.3 mg/kg (1.7 ± 0.2 mg/kg for men and 2.1 ± 0.3 mg/kg for women; range: 1.4–2.7 mg/kg).

### Genotyping

Genomic DNA was extracted from whole blood using the QIAamp DNA Blood Mini Kit (Qiagen, Hombrechtikon, Switzerland) and automated QIAcube system. Genotyping was performed using commercial TaqMan SNP genotyping assays (LuBio Science, Lucerne, Switzerland). The *OXTR* is located on the short arm of chromosome 3 (3p25) and has three introns and four exons. We genotyped three OXTR SNPs: *rs53576* (c.922+4581T>C, position [GRCh37]: chr3:8804371, assay: C___3290335_10), *rs1042778* (c.*118C>A, position [GRCh37]: chr3:8794545, assay: C___7622140_30), and *rs2254298* (c.922+6724C>T, position [GRCh37]: chr3:8802228, assay: C__15981334_10). Linkage disequilibrium analysis between the SNPs were performed using Haploview (Broad Institute).

### Subjective effects

Visual Analog Scales (VASs) were repeatedly applied to assess subjective effects over time [1]. The VAS “any drug effect” was presented as 100 mm horizontal lines (0–100%), marked from “not at all” on the left to “extremely” on the right. The VASs “closeness to others,” “trust,” “want to be hugged,” “want to hug,” “want to be alone,” and “want to be with others” were bidirectional (±50%). “Trust,” “want to be hugged,” “want to hug,” “want to be alone,” and”want to be with others” were assessed in 53 subjects. The VASs were administered before and 0, 0.33, 0.67, 1, 1.5, 2, 2.5, 3, 4, 5, and 6 h after MDMA or placebo administration.

### Emotion recognition

To measure emotion recognition, we used the Facial Emotion Recognition Task (FERT), which is sensitive to the effects of MDMA [3, 5, 12, 43] and other serotonergic substances [48]. The task included 10 neutral faces and 160 faces that expressed one of four basic emotions (i.e., happiness, sadness, anger, and fear), with pictures morphed between 0% (neutral) and 100% in 10% steps. Two female and two male pictures were used for each of the four emotions. The stimuli were presented in random order for 500 ms and then were replaced by the rating screen where the participants had to indicate the correct emotion. The outcome measure was accuracy (proportion correct) and misclassification (emotions that were indicated incorrectly). The FERT was performed 90 min after drug administration. FERT data were available from 69 subjects. The genotype distribution for this subsample was: *rs1042778* (28 GG, 33 GT, 8 TT), *rs53576* (30 GG, 25 AG, 14 AA), and *rs2254298* (57 GG, 12 AG/AA).

### Empathy

The Multifaceted Empathy Test (MET) is a reliable and valid task that assesses the cognitive and emotional aspects of empathy [49]. The MET is sensitive to oxytocin [17], MDMA [1, 3, 20], and other psychoactive substances [48]. The computer-assisted test consisted of 40 photographs that showed people in emotionally charged situations. To assess cognitive empathy, the participants were required to infer the mental state of the subject in each scene and indicate the correct mental state from a list of four responses. Cognitive empathy was defined as the percentage of correct responses relative to total responses. To measure emotional empathy, the subjects were asked to rate how much they were feeling for an individual in each scene (i.e., explicit emotional empathy) and how much they were aroused by each scene (i.e., implicit emotional empathy) on a 1–9 point scale. The latter rating provides an inherent additional assessment of emotional empathy, which is considered to reduce the likelihood of socially desirable answers. The three aspects of empathy were each tested with 20 stimuli with positive valence and 20 stimuli with negative valence, resulting in a total of 120 trials. The MET was performed 90–180 min after drug administration. MET data were available from 69 subjects. The genotype distribution for this subsample was: *rs1042778* (28 GG, 33 GT, 8 TT), *rs53576* (30 GG, 25 AG, 14 AA), and *rs2254298* (57 GG, 12 AG/AA).

### Prosociality

We used the paper version of the validated Social Value Orientation (SVO) test to assess social behavior [50]. The SVO test is sensitive to MDMA [1] and other psychoactive substances [48]. In this economic resource allocation task, prosociality is defined as behavior that maximizes the sum of resources for the self and others and minimizes the difference between the two. The test consists of six primary and nine secondary SVO slider items with a resource allocation choice over a defined continuum of joint payoffs [50]. The participants were instructed to choose a resource allocation that defined their most preferred joint distribution between themselves and another person. The allocated funds had real value, and four randomly selected subjects received the funds they earned. Mean allocations for the self and the other were calculated [1, 50], and the inverse tangent of the ratio of these two means produced an angle that indicated the participants’ SVO index. A smaller SVO angle indicates more individualistic or competitive behavior, and a larger SVO angle indicates more prosocial or even altruistic behavior. The nine secondary items were used to differentiate between two different prosocial motivations (inequality aversion and joint gain maximization). The inequality-aversion index was calculated as previously described [50]. An index of 0 indicates perfect inequality aversion, and 1 indicates maximal preference for joint gain maximization. The SVO test was performed 3–4 h after drug administration. SVO primary data and the inequality-aversion index were available from 69 and 33 subjects, respectively. The genotype distribution for this subsample was: *rs1042778* (28 GG, 33 GT, 8 TT), *rs53576* (30 GG, 25 AG, 14 AA), and *rs2254298* (57 GG, 12 AG/AA) and *rs1042778* (15 GG, 15 GT, 3 TT), *rs53576* (12 GG, 15 AG, 6 AA), and *rs2254298* (26 GG, 7 AG/AA), respectively.

### Plasma concentrations of oxytocin and MDMA

The plasma concentration of oxytocin has been shown to peak 2 h after MDMA administration [10] and was therefore measured at baseline and 2 h after drug administration and analyzed as described previously [4, 23, 51]. The plasma level of MDMA was determined 1 h before and 0.5, 1, 1.5, 2, 3, 4, and 6 h after drug administration and analyzed as described previously [8].

### Statistical analysis

Subjective drug effects on the VASs were determined as the area under the effect-time curve from 0 to 6 h (AUEC_6_) after drug administration using the trapezoidal method in Phoenix WinNonlin (version 6.4, Pharsight, Certara L.P., St. Louis, MO, USA). The statistical analyses were performed using Statistica 12 software (StatSoft, Tulsa, OK, USA). The effects of MDMA on subjective effect ratings and plasma oxytocin concentrations were expressed as differences from placebo. Repeated-measured analyses of variance (ANOVAs), with drug (MDMA *vs*. placebo) as the within-subjects factor, were used to evaluate drug effects. One-way ANOVAs, with genotype group as the between-subjects factor, followed by the Tukey post hoc test were used to evaluate the effects of genotype on the effects of MDMA (differences from placebo). Additional ANOVAs including plasma concentrations of MDMA and/or oxytocin as covariates were conducted as well as sex differences were included by adding sex as an additional between-subjects factor to the analyses to exclude confounding by any of these variables. This showed that the results were not confounded by oxytocin plasma levels, or sex, or differences in plasma concentrations of MDMA, that corrects for differences in body weight, dosing, or/and known and unknown activity differences in metabolizing enzymes [46, 47]. The reported results are from analysis with MDMA plasma concentration AUC_6_ and oxytocin plasma concentration change at 2 h as covariates with the exception of the VAS “any drug effect” and “closeness to others” for which only MDMA plasma concentration AUC_6_ was corrected due to the lack of oxytocin plasma concentration data for subjects from the first two studies [8, 41]. The level of significance was set to p < 0.05. The Nyholt correction method was used to account for multiple comparisons and flagged separately [52]. We thereby corrected for the 7 VASs, 3 emotions in the FERT, emotional empathy in the MET, and 2 items in the SVO which have all been shown sensitive to the effects of MDMA and for each of the 3 tested SNPs ([7+3+1+2] × 3), resulting in 39 variables and an effective number of independent variables (V_eff_) of 28.96 according to Nyholt and a corrected significance threshold to keep Type I error rate at 5% of p < 0.0017. For the analysis of the *rs2254298* SNP, the AA and AG genotypes were pooled as A allele carriers because only one subject had the AA genotype.

## Results

### Genotyping

The distribution of the alleles and genotypes did not differ from distributions reported elsewhere in Caucasian cohorts (Ensembl database release 86, Oct 2016). The rare allele frequencies for *rs53576*, *rs1042778*, and *rs2254298* were A (93 [35%]), T (101 [38%]), and A (20 [11%]), respectively. No linkage disequilibrium was detected between the tested SNPs (S1 Fig).

### Subjective effects

**Fig 1:**
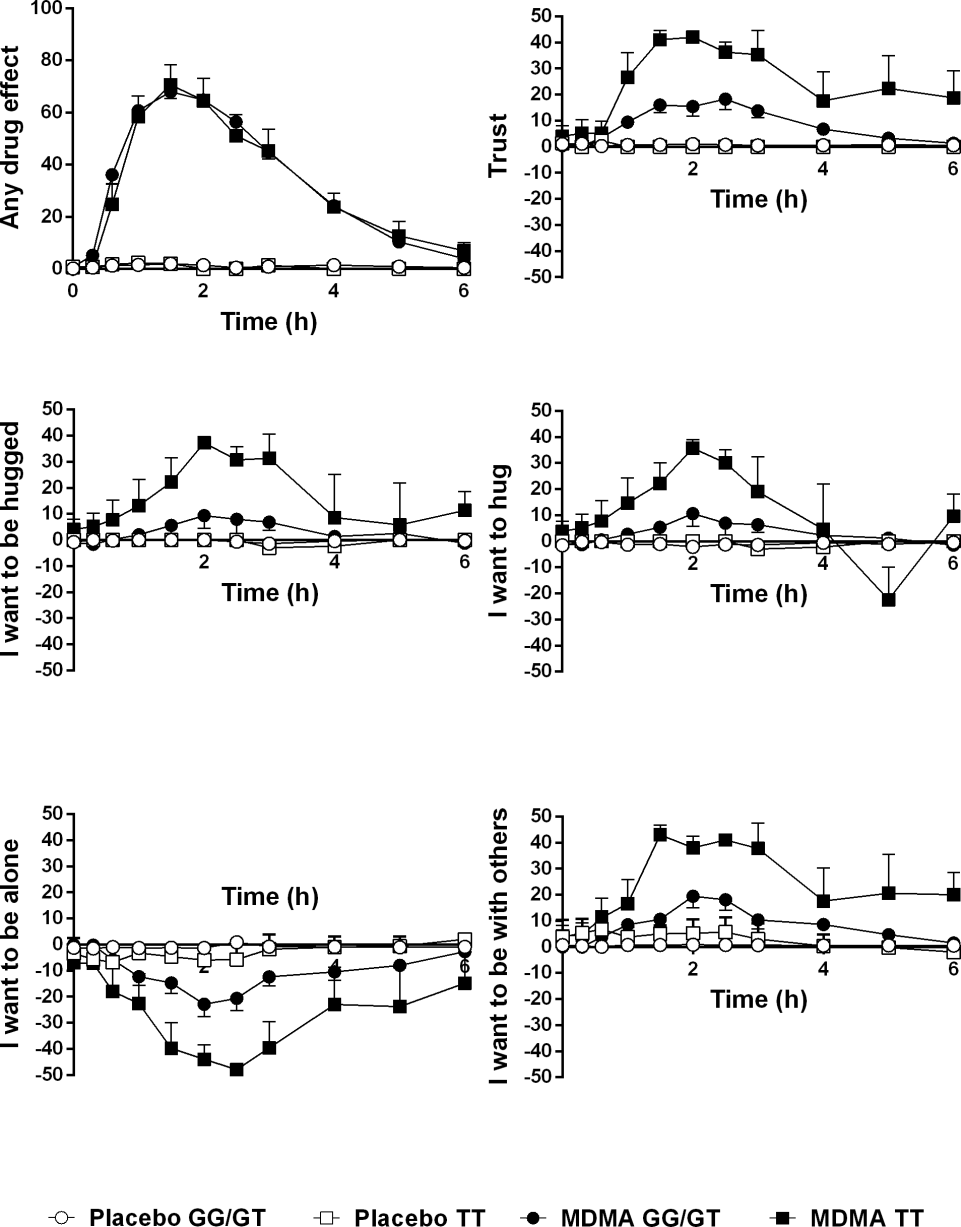
Effect of the OXTR rs1042778 SNP on the subjective effects of MDMA. MDMA produced greater “trust,” “want to be hugged,” “want to hug,” and “want to be with others” and reduced “want to be alone” in the TT group (n = 5) compared with the GG/GT group (n = 48, Table 1 and S1 Table). The data are expressed as mean ± SEM. MDMA or placebo was administered at time = 0.

On the VASs, MDMA increased the AUEC_6_ values for “any drug effect” (*F*_1,131_ = 544, *p* < 0.001), “closeness” (*F*_1,131_ = 57, *p* < 0.001), “trust” (*F*_1,52_ = 33; *p* < 0.001), “want to be hugged” (*F*_1,52_ = 6.6, *p* < 0.05), “want to hug” (*F*_1,52_ = 7.6, *p* < 0.01), and “want to be with others” (*F*_1,52_ = 20, *p* < 0.001) and decreased feelings of “want to be alone” (*F*_1,52_ = 21, *p* < 0.001, Fig 1, Table 1).

**Table 1:** Effects of oxytocin receptor rs53576, rs1042778, and rs2254298 polymorphism groups on the response to MDMA (mean±SD and statistics).

		SNP rs1042778					SNP rs53576					SNP rs2254298				
		GG/GT	TT	F	p value	p value^a^	GA/GG	AA	F	p value	p value^a^	GG	AA/AG	F	p value	p value^a^
	N (%)	113 (86)	19 (14)				113 (86)	19 (14)				105 (80)	27 (20)			
	Male, N (%)	57 (50)	7 (37)				52 (46)	12 (63)				51 (49)	13 (48)			
	MDMA plasma concentration AUC_6_ (ng*h/mL)	954±212	924±179	0.34	NS	NS	956±205	914±219	0.68	NS	NS	941±208	985±200	0.99	NS	NS
	MDMA peak concentrations (ng/mL)	225±49	223±41	0.02	NS	NS	226±47	214±53	1	NS	NS	222±47	235±50	1.8	NS	NS
	Oxytocin Δplasma concentration at 2h (pg/mL)^b^	53±63	64±101	0.33	NS	NS	55±71	53±60	0.01	NS	NS	53±68	63±74	0.35	NS	NS
Visual Analog Scale rating ΔAUEC_6_																
	Any drug effect	195±95	196±107	0.16	NS	NS	193±98	205±88	1.14	NS	NS	188±95	221±97	1.55	NS	NS
	Closeness to others	37±57	64±87	3.89	0.051	NS	39±60	54±74	1.59	NS	NS	41±65	41±51	0.07	NS	NS
	Trust^c^	46±64	153±65	14.03	<0.001	0.014	58±75	52±59	0.02	NS	NS	50±72	86±60	1.06	NS	NS
	Want to be hugged^c^	20±68	105±129	5.27	0.026	NS	29±80	23±78	0.03	NS	NS	26±82	37±59	0.02	NS	NS
	Want to hug^c^	22±65	63±101	1.53	NS	NS	29±70	18±66	0.01	NS	NS	26±72	26±57	0.15	NS	NS
	Want to be alone^c^	-55±97	-153±108	4.49	0.039	NS	-55±96	-91±116	2.62	NS	NS	-58±105	-93±78	0.28	NS	NS
	Want to be with others^c^	43±77	140±120	7.28	0.010	NS	47±85	67±88	1.56	NS	NS	48±91	70±45	0.09	NS	NS

N, number of subjects; AUEC, area under the effect-time curve; SD, standard deviation; NS, not significant; Δ, values are change scores from placebo.

^a^p value additionally corrected for multiple comparisons according to the Nyholt correction.

^b^N = 101 (rs1042778: 87 GG/GT, 14 TT; rs53576: 83 GG/AG, 18 AA; rs2254298: 81 GG, 20 AG/AA).

^c^N = 53 (rs1042778: 48 GG/GT, 5 TT; rs53576: 40 GG/AG, 13 AA; rs2254298: 44 GG, 9 AG/AA).

The effects of the OXTR *rs1042778* SNP on the subjective effects of MDMA are shown in Table 1, Fig 1, and S1 Table. MDMA produced increases in “trust” (*F*_1,49_ = 14, *p* < 0.001), “want to be hugged” (*F*_1,49_ = 5.3, *p* < 0.05), and “want to be with others” (*F*_1,49_ = 6.5, *p* < 0.05) in the TT genotype group compared with the G allele carriers. MDMA lowered ratings of “wanting to be alone” more in subjects with the TT genotype compared with G allele carriers (*F*_1,49_ = 4.5, *p* < 0.05). Using Nyholt correction for the multiple comparisons, only MDMA effects on “trust” was significantly altered by the *rs1042778* SNP. The OXTR *rs53576* and *rs2254298* SNPs did not alter the subjective effects of MDMA (Table 1).

### Emotion recognition

On the FERT, MDMA impaired the recognition of fearful (*F*_1,68_ = 47, *p* < 0.001), sad (*F*_1,68_ = 14, *p* < 0.001), and angry (*F*_1,68_ = 16, *p* < 0.001) faces compared with placebo. None of the OXTR gene variants moderated the effects of MDMA on the FERT.

### Empathy

MDMA increased explicit emotional empathy for positive emotions (*F*_1,68_ = 7.6, *p* < 0.01) compared with placebo. None of the OXTR gene variants altered the effects of MDMA on the MET.

### Prosociality

MDMA produced a near-significant trend toward an increase in the SVO angle compared with placebo (*F*_1,68_ = 3.1, *p* = 0.08). MDMA reduced the inequality-aversion index (*F*_1,32_ = 9.3, *p* < 0.01) in subjects with a prosocial orientation, indicating a shift from joint gain maximization to inequality aversion. The *rs53576* and *rs1042778* SNPs moderated the MDMA-induced increase in inequality aversion (S2 Fig). MDMA significantly reduced the inequality-aversion index in the *rs53576* AA genotype group compared with G allele carriers (*F*_1,31_ = 9.4, *p* < 0.01). MDMA also reduced inequality-aversion in the *rs1042778* GG genotype group compared with T allele carriers (*F*_1,29_ = 5.6, *p* < 0.05). T allele carriers who received placebo also had a lower inequality-aversion index (corresponding to greater inequality-aversion) compared with subjects with the GG genotype (*p* < 0.05). However, if corrected for multiple comparisons none of the OXTR genetics influence SVO findings significantly.

### Plasma concentrations of oxytocin and MDMA

Plasma oxytocin and MDMA concentrations are shown in Table 1. Oxytocin concentrations were significantly elevated 2 h after MDMA administration compared with placebo (placebo: 19 ± 39 pg/ml; MDMA: 74 ± 70 pg/ml; *F*_1,99_ = 62, *p* < 0.001). Plasma oxytocin and MDMA concentrations similarly increased across all OXTR gene variants (Table 1). MDMA peak concentrations and AUC_6_ values were (mean ± SD) 224 ± 48 ng/mL and 950 ± 207 ng×h/mL in the total of 132 subjects. There was acute tolerance to the subjective response to MDMA.

## Discussion

The main finding of this study was that the OXTR *rs1042778* SNP influenced the typical empathogenic and prosocial feelings that are produced by MDMA, including enhancements of “trust”. Similar modulation of the prosocial effects of MDMA was previously reported for the OXTR *rs53576* SNP [30] but not *rs1042778* SNP. These findings suggest that humans may respond differently to the typical subjective effects of MDMA, depending on their OXTR genetics. The results indirectly indicate a possible role for oxytocin in the subjective effects of MDMA, similar to its interoceptive effects in rats [53]. Animal studies have shown that oxytocin mediates the prosocial effects of MDMA [13, 14, 54]. We observed lower subjective prosociality after MDMA administration in carriers of the G allele of the *rs1042778* SNP. Greater prosociality [33] and lower antisocial behavior [55] have been associated with the G allele in the absence of treatment. Our placebo condition was unsuitable for assessing differences in prosociality between subjects in the absence of treatment. Altogether, however, the findings are consistent with the notion that subjects with lower sociality may respond more to the prosocial effects of MDMA or oxytocin [56].

In the present study, we failed to replicate a previous finding of moderation of the subjective prosocial effects of MDMA by the *rs53576* SNP [30]. This previous study showed that carriers of the AA genotype at the *rs53576* locus were not susceptible to the prosocial effect of 1.5 mg/kg MDMA [30]. However, this previous study assessed “sociability” as a combined outcome of several VASs, including “friendly,” “sociable,” “confident,” “playful,” and “loving” using principal component analysis [30]. Additionally, the effect of *rs53576* was observed only at a dose of 1.5 mg/kg MDMA, whereas opposite effects were observed with 0.75 mg/kg MDMA [30]. The discrepant findings between these two studies may be partially explained by the different scales that were used. The findings may also indicate that the MDMA effect modulation by different OXTR SNPs is not very robust across studies and rather small.

The effects of MDMA on the FERT and MET in the present study were consistent with studies by other researchers who used the same tests [5, 20]. The present study found that the *rs53576*, *rs1042778*, and *rs2254298* SNPs did not influence MDMA-induced impairments in the recognition of fearful, sad, and angry faces or increases in emotional empathy. No other data are available on the effects of other OXTR gene variants on MDMA-induced changes in tests of emotion processing or empathy. In contrast to the present study, the effect of intranasal oxytocin on dynamic face recognition has previously been shown to be modulated by OXTR SNP haplotypes, including the SNPs that were studied herein [32].

On the SVO test, MDMA produced a trend toward an increase in prosocial behavior and increased inequality aversion. We previously reported a significant increase in prosociality and a trend toward an increase in inequality aversion from a subset of the present data [1]. A novel finding of the present study was that MDMA increased inequality-aversion and thus a preference for fairness only in subjects with the *rs53576* AA or *rs1042778* GG genotypes, indicating a role for these OXTR gene variants in MDMA’s effect on social behavior. However, the MDMA-induced increase in preference for fairness in *rs1042778* GG subjects appears to conflict with the smaller increase in trust in these subjects (S1 Table). Additionally, *rs1042778* GG individuals presented lower inequality aversion compared with T allele carriers who received placebo. Higher prosociality on the SVO test has previously been reported in G allele carriers [33], but differences in the inequality-aversion index were not studied because this scale was only added later to the SVO test [50]. Furthermore, the OXTR *rs1042778*, *rs53576*, and *rs2254298* SNPs had no effects in two other economic games (i.e., dictator game and trust game; [57]). The findings in the SVO tests are conclusive but need to be interpreted with caution, since they did not survive the correction for multiple comparisons and the total number of subjects in this subset was reduced to 33 due to the limiting calculation of the inequality aversion [50].

The present study has several limitations. First, the study was mostly exploratory and the findings would need to be confirmed in larger studies. Second, not all outcome measures were used in all of the subjects, thus limiting the sample size and also increasing the risk of Type I errors. Third, we tested only three OXTR SNPs. Other SNPs or haplotypes may also play a role [32]. Fourth, MDMA causes the release of oxytocin, monoamines [8, 9], and arginine vasopressin [51]. The latter two are well known to influence social cognition and behavior [58]. MDMA also increases cortisol and other corticosteroids [59, 60] and oxytocin and cortisol may interact to influence the response to MDMA and these interactions need further study [21]. Finally, cultural and early environmental background plays an uncertain role in the results of genetic studies, especially studies of OXTRs. For example, studies of the *rs2254298* SNP reported different results in Caucasian and Asian subjects [35, 61].

The present findings of individual differences in the response to MDMA that depended on OXTR genetics need to be confirmed and might have implications for MDMA-assisted psychotherapy [7] and may contribute to more personalized treatment. Therapeutic studies that use MDMA in patients should genotype OXTR SNPs and test for polymorphisms of the genes that regulate the metabolism of MDMA [46, 47].

## Conclusion

The OXTR *rs1042778* SNP but not the *rs53576* or *rs2254298* SNPs altered the typical MDMA-induced feelings of trust. A previous finding of a moderating influence of the rs53576 on the socio-emotional effect of MDMA could not be replicated indicating a chance finding. Additionally, after correction for multiple comparisons OXTR SNPs did not moderate the subjective overall effect of MDMA (any drug effect) or feelings of “closeness to others” in the total larger study sample of 132 subjects. Thus, the results are preliminary and should be interpreted with caution due to multiple comparisons and small genotype group sample sizes.

## Supporting information

S1 Fig Linkage disequilibrium across the determined SNPs. Estimates of the square of the correlation coefficient (r^2^) were calculated for each pairwise comparison of SNPs based on data from our study cohort.
(TIF)

S2 Fig OXTR rs53576 and rs104278 SNPs moderate the effects of MDMA on the SVO Inequality-aversion index. **(a)** MDMA reduced the inequality-aversion index in the OXTR rs53576 AA genotype group (n = 6) but not in the GG/GA genotype group (n = 27, **p < 0.01). **(b)** MDMA reduced the inequality-aversion index in the OXTR rs1042778 GG genotype group (n = 15) but not in the TT/TG genotype group (n = 18, *p < 0.05, **p < 0.01). The data are expressed as mean ± SEM. If corrected for multiple comparisons none of the OXTR genetics significantly influence SVO findings. An inequality-aversion index of 0 indicates perfect inequality aversion (maximal fairness), and 1 indicates maximal preference for joint gain maximization in subjects with a prosocial value orientation.
(TIF)

S1 Table Effects of oxytocin receptor rs53576 and rs1042778 polymorphisms (all allele groups) on the response to MDMA (mean±SD and statistics). N, number of subjects; AUEC, area under the effect-time curve; SD, standard deviation; NS, not significant; D, values are change scores from placebo; **p value < 0.01 compared to rs1042778 TT. ^a^p value additionally corrected for multiple comparisons according to the Nyholt correction. ^b^N = 101 (rs1042778: 39 GG, 48 GT, 14 TT; rs53576: 44 GG, 39 AG, 18 AA; rs2254298: 80 GG, 21 AG/AA). ^c^N = 53 (rs1042778: 21 GG, 27 GT, 5 TT; rs53576: 23 GG, 17 AG, 13 AA; rs2254298: 44 GG, 9 AG/AA).
(XLSX)

## References

[1] Hysek CM, Schmid Y, Simmler LD, Domes G, Heinrichs M, Eisenegger C, et al MDMA enhances emotional empathy and prosocial behavior. Soc Cogn Affect Neurosci. 2014;9:1645–52. doi: 10.1093/scan/nst161 24097374 10.1093/scan/nst161 PMC4221206

[2] Wardle MC, Kirkpatrick MG, de Wit H. 'Ecstasy' as a social drug: MDMA preferentially affects responses to emotional stimuli with social content. Soc Cogn Affect Neurosci. 2014;9:1076–81. doi: 10.1093/scan/nsu035 24682132 10.1093/scan/nsu035 PMC4127030

[3] Schmid Y, Hysek CM, Simmler LD, Crockett MJ, Quednow BB, Liechti ME. Differential effects of MDMA and methylphenidate on social cognition. J Psychopharmacol. 2014;28:847–56. doi: 10.1177/0269881114542454 25052243 10.1177/0269881114542454

[4] Hysek CM, Domes G, Liechti ME. MDMA enhances "mind reading" of positive emotions and impairs "mind reading" of negative emotions. Psychopharmacology (Berl). 2012;222:293–302. 22277989 10.1007/s00213-012-2645-9

[5] Bedi G, Hyman D, de Wit H. Is ecstasy an "empathogen"? Effects of ±3,4-methylenedioxymethamphetamine on prosocial feelings and identification of emotional states in others. Biol Psychiatry. 2010;68:1134–40. doi: 10.1016/j.biopsych.2010.08.003 20947066 10.1016/j.biopsych.2010.08.003 PMC2997873

[6] Kuypers KPC, Dolder PC, Ramaekers JG, Liechti ME. Multifaceted empathy of healthy volunteers after single doses of MDMA: a pooled sample of placebo-controlled studies. J Psychopharmacol. 2017;31:589–98. doi: 10.1177/0269881117699617 28372480 10.1177/0269881117699617 PMC5418931

[7] Mithoefer MC, Wagner MT, Mithoefer AT, Jerome I, Doblin R. The safety and efficacy of ±3,4-methylenedioxymethamphetamine-assisted psychotherapy in subjects with chronic, treatment-resistant posttraumatic stress disorder: the first randomized controlled pilot study. J Psychopharmacol. 2010;25:439–52. doi: 10.1177/0269881110378371 20643699 10.1177/0269881110378371 PMC3122379

[8] Hysek CM, Simmler LD, Nicola V, Vischer N, Donzelli M, Krähenbühl S, et al Duloxetine inhibits effects of MDMA ("ecstasy") in vitro and in humans in a randomized placebo-controlled laboratory study. PLoS One. 2012;7:e36476 doi: 10.1371/journal.pone.0036476 22574166 10.1371/journal.pone.0036476 PMC3344887

[9] Simmler L, Buser T, Donzelli M, Schramm Y, Dieu LH, Huwyler J, et al Pharmacological characterization of designer cathinones in vitro. Br J Pharmacol. 2013;168:458–70. doi: 10.1111/j.1476-5381.2012.02145.x 22897747 10.1111/j.1476-5381.2012.02145.x PMC3572571

[10] Dumont GJ, Sweep FC, van der Steen R, Hermsen R, Donders AR, Touw DJ, et al Increased oxytocin concentrations and prosocial feelings in humans after ecstasy (3,4-methylenedioxymethamphetamine) administration. Soc Neurosci. 2009;4:359–66. doi: 10.1080/17470910802649470 19562632 10.1080/17470910802649470

[11] Francis SM, Kirkpatrick MG, de Wit H, Jacob S. Urinary and plasma oxytocin changes in response to MDMA or intranasal oxytocin administration. Psychoneuroendocrinology. 2016;74:92–100. doi: 10.1016/j.psyneuen.2016.08.011 27592327 10.1016/j.psyneuen.2016.08.011

[12] Kirkpatrick MG, Lee R, Wardle MC, Jacob S, de Wit H. Effects of MDMA and intranasal oxytocin on social and emotional processing. Neuropsychopharmacology. 2014;39:1654–63. doi: 10.1038/npp.2014.12 24448644 10.1038/npp.2014.12 PMC4023138

[13] Ramos L, Hicks C, Caminer A, Couto K, Narlawar R, Kassiou M, et al MDMA ('Ecstasy'), oxytocin and vasopressin modulate social preference in rats: A role for handling and oxytocin receptors. Pharmacol Biochem Behav. 2016;150–151:115–23. doi: 10.1016/j.pbb.2016.10.002 27725273 10.1016/j.pbb.2016.10.002

[14] Thompson MR, Callaghan PD, Hunt GE, Cornish JL, McGregor IS. A role for oxytocin and 5-HT_1A_ receptors in the prosocial effects of 3,4 methylenedioxymethamphetamine ("ecstasy"). Neuroscience. 2007;146:509–14. doi: 10.1016/j.neuroscience.2007.02.032 17383105 10.1016/j.neuroscience.2007.02.032

[15] Wardle MC, de Wit H. MDMA alters emotional processing and facilitates positive social interaction. Psychopharmacology (Berl). 2014;231:4219–29. 24728603 10.1007/s00213-014-3570-x PMC4194242

[16] Di Simplicio M, Massey-Chase R, Cowen PJ, Harmer CJ. Oxytocin enhances processing of positive versus negative emotional information in healthy male volunteers. J Psychopharmacol. 2009;23:241–8. doi: 10.1177/0269881108095705 18801829 10.1177/0269881108095705

[17] Hurlemann R, Patin A, Onur OA, Cohen MX, Baumgartner T, Metzler S, et al Oxytocin enhances amygdala-dependent, socially reinforced learning and emotional empathy in humans. J Neurosci. 2010;30:4999–5007. doi: 10.1523/JNEUROSCI.5538-09.2010 20371820 10.1523/JNEUROSCI.5538-09.2010 PMC6632777

[18] Kosfeld M, Heinrichs M, Zak PJ, Fischbacher U, Fehr E. Oxytocin increases trust in humans. Nature. 2005;435:673–6. doi: 10.1038/nature03701 15931222 10.1038/nature03701

[19] Ditzen B, Schaer M, Gabriel B, Bodenmann G, Ehlert U, Heinrichs M. Intranasal oxytocin increases positive communication and reduces cortisol levels during couple conflict. Biol Psychiatry. 2009;65:728–31. doi: 10.1016/j.biopsych.2008.10.011 19027101 10.1016/j.biopsych.2008.10.011

[20] Kuypers KP, de la Torre R, Farre M, Yubero-Lahoz S, Dziobek I, Van den Bos W, et al No evidence that MDMA-induced enhancement of emotional empathy is related to peripheral oxytocin levels or 5-HT1a receptor activation. PLoS One. 2014;9:e100719 doi: 10.1371/journal.pone.0100719 24972084 10.1371/journal.pone.0100719 PMC4074089

[21] Parrott AC. Oxytocin, cortisol and 3,4-methylenedioxymethamphetamine: neurohormonal aspects of recreational 'ecstasy'. Behav Pharmacol. 2016;27:649–58. doi: 10.1097/FBP.0000000000000262 27681116 10.1097/FBP.0000000000000262

[22] Broadbear JH, Kabel D, Tracy L, Mak P. Oxytocinergic regulation of endogenous as well as drug-induced mood. Pharmacol Biochem Behav. 2014;119:61–71. doi: 10.1016/j.pbb.2013.07.002 23872370 10.1016/j.pbb.2013.07.002

[23] Neumann ID, Maloumby R, Beiderbeck DI, Lukas M, Landgraf R. Increased brain and plasma oxytocin after nasal and peripheral administration in rats and mice. Psychoneuroendocrinology. 2013;38:1985–93. doi: 10.1016/j.psyneuen.2013.03.003 23579082 10.1016/j.psyneuen.2013.03.003

[24] Parrott AC. The psychotherapeutic potential of MDMA (3,4-methylenedioxymethamphetamine): an evidence-based review. Psychopharmacology (Berl). 2007;191:181–93. 17297639 10.1007/s00213-007-0703-5

[25] Parrott AC, Gibbs A, Scholey AB, King R, Owens K, Swann P, et al MDMA and methamphetamine: some paradoxical negative and positive mood changes in an acute dose laboratory study. Psychopharmacology (Berl). 2011;215:527–36. 21318566 10.1007/s00213-011-2184-9

[26] Vizeli P, Liechti ME. Safety pharmacology of acute MDMA administration in healthy subjects. J Psychopharmacol. 2017;31:576–88. doi: 10.1177/0269881117691569 28443695 10.1177/0269881117691569

[27] Liechti ME, Gamma A, Vollenweider FX. Gender differences in the subjective effects of MDMA. Psychopharmacology (Berl). 2001;154:161–8. 11314678 10.1007/s002130000648

[28] Feldman R, Monakhov M, Pratt M, Ebstein RP. Oxytocin pathway genes: evolutionary ancient system impacting on human affiliation, sociality, and psychopathology. Biol Psychiatry. 2016;79:174–84. doi: 10.1016/j.biopsych.2015.08.008 26392129 10.1016/j.biopsych.2015.08.008

[29] Kumsta R, Heinrichs M. Oxytocin, stress and social behavior: neurogenetics of the human oxytocin system. Curr Opin Neurobiol. 2013;23:11–6. doi: 10.1016/j.conb.2012.09.004 23040540 10.1016/j.conb.2012.09.004

[30] Bershad AK, Weafer JJ, Kirkpatrick MG, Wardle MC, Miller MA, de Wit H. Oxytocin receptor gene variation predicts subjective responses to MDMA. Soc Neurosci. 2016;11:592–9. doi: 10.1080/17470919.2016.1143026 26787430 10.1080/17470919.2016.1143026 PMC4988944

[31] Rodrigues SM, Saslow LR, Garcia N, John OP, Keltner D. Oxytocin receptor genetic variation relates to empathy and stress reactivity in humans. Proc Natl Acad Sci U S A. 2009;106:21437–41. doi: 10.1073/pnas.0909579106 19934046 10.1073/pnas.0909579106 PMC2795557

[32] Chen FS, Kumsta R, Dvorak F, Domes G, Yim OS, Ebstein RP, et al Genetic modulation of oxytocin sensitivity: a pharmacogenetic approach. Transl Psychiatry. 2015;5:e664 doi: 10.1038/tp.2015.163 26506050 10.1038/tp.2015.163 PMC4930136

[33] Israel S, Lerer E, Shalev I, Uzefovsky F, Riebold M, Laiba E, et al The oxytocin receptor (OXTR) contributes to prosocial fund allocations in the dictator game and the social value orientations task. PLoS One. 2009;4:e5535 doi: 10.1371/journal.pone.0005535 19461999 10.1371/journal.pone.0005535 PMC2680041

[34] Feldman R, Zagoory-Sharon O, Weisman O, Schneiderman I, Gordon I, Maoz R, et al Sensitive parenting is associated with plasma oxytocin and polymorphisms in the OXTR and CD38 genes. Biol Psychiatry. 2012;72:175–81. doi: 10.1016/j.biopsych.2011.12.025 22336563 10.1016/j.biopsych.2011.12.025

[35] Wu S, Jia M, Ruan Y, Liu J, Guo Y, Shuang M, et al Positive association of the oxytocin receptor gene (OXTR) with autism in the Chinese Han population. Biol Psychiatry. 2005;58:74–7. doi: 10.1016/j.biopsych.2005.03.013 15992526 10.1016/j.biopsych.2005.03.013

[36] Costa B, Pini S, Gabelloni P, Abelli M, Lari L, Cardini A, et al Oxytocin receptor polymorphisms and adult attachment style in patients with depression. Psychoneuroendocrinology. 2009;34:1506–14. doi: 10.1016/j.psyneuen.2009.05.006 19515497 10.1016/j.psyneuen.2009.05.006

[37] LoParo D, Waldman ID. The oxytocin receptor gene (OXTR) is associated with autism spectrum disorder: a meta-analysis. Mol Psychiatry. 2015;20:640–6. doi: 10.1038/mp.2014.77 25092245 10.1038/mp.2014.77

[38] Watanabe T, Otowa T, Abe O, Kuwabara H, Aoki Y, Natsubori T, et al Oxytocin receptor gene variations predict neural and behavioral response to oxytocin in autism. Soc Cogn Affect Neurosci. 2017;12:496–506. doi: 10.1093/scan/nsw150 27798253 10.1093/scan/nsw150 PMC5390696

[39] Hysek CM, Brugger R, Simmler LD, Bruggisser M, Donzelli M, Grouzmann E, et al Effects of the alpha_2_-adrenergic agonist clonidine on the pharmacodynamics and pharmacokinetics of 3,4-methylenedioxymethamphetamine in healthy volunteers. J Pharmacol Exp Ther. 2012;340:286–94. doi: 10.1124/jpet.111.188425 22034656 10.1124/jpet.111.188425

[40] Hysek CM, Schmid Y, Rickli A, Simmler LD, Donzelli M, Grouzmann E, et al Carvedilol inhibits the cardiostimulant and thermogenic effects of MDMA in humans. Br J Pharmacol. 2012;166:2277–88. doi: 10.1111/j.1476-5381.2012.01936.x 22404145 10.1111/j.1476-5381.2012.01936.x PMC3448893

[41] Hysek CM, Simmler LD, Ineichen M, Grouzmann E, Hoener MC, Brenneisen R, et al The norepinephrine transporter inhibitor reboxetine reduces stimulant effects of MDMA ("ecstasy") in humans. Clin Pharmacol Ther. 2011;90:246–55. doi: 10.1038/clpt.2011.78 21677639 10.1038/clpt.2011.78

[42] Hysek CM, Liechti ME. Effects of MDMA alone and after pretreatement with reboxetine, duloxetine, clonidine, carvedilol, and doxazosin on pupillary light reflex. Psychopharmacology (Berl). 2012;224:363–76. 22700038 10.1007/s00213-012-2761-6

[43] Hysek CM, Simmler LD, Schillinger N, Meyer N, Schmid Y, Donzelli M, et al Pharmacokinetic and pharmacodynamic effects of methylphenidate and MDMA administered alone and in combination. Int J Neuropsychopharmacol. 2014;17:371–81. doi: 10.1017/S1461145713001132 24103254 10.1017/S1461145713001132

[44] Dolder PC, Muller F, Schmid Y, Borgwardt SJ, Liechti ME. Direct comparison of the acute subjective, emotional, autonomic, and endocrine effects of MDMA, methylphenidate, and modafinil in healthy subjects. Psychopharmacology (Berl). 2017; 235(2):467–479. 28551715 10.1007/s00213-017-4650-5 PMC5813072

[45] Schmid Y, Rickli A, Schaffner A, Duthaler U, Grouzmann E, Hysek CM, et al Interactions between bupropion and 3,4-methylenedioxymethamphetamine in healthy subjects. J Pharmacol Exp Ther. 2015;353:102–11. doi: 10.1124/jpet.114.222356 25655950 10.1124/jpet.114.222356

[46] Schmid Y, Vizeli P, Hysek CM, Prestin K, Meyer zu Schwabedissen HE, Liechti ME. CYP2D6 function moderates the pharmacokinetics and pharmacodynamics of MDMA in a controlled study in healthy subjects. Pharmacogenet Genom. 2016;26:397–401. 10.1097/FPC.0000000000000231 PMC4949007 27253829

[47] Vizeli P, Schmid Y, Prestin K, Meyer zu Schwabedissen HE, Liechti ME. Pharmacogenetics of ecstasy: CYP1A2, CYP2C19, and CYP2B6 polymorphisms moderate pharmacokinetics of MDMA in healthy subjects. Eur Neuropsychopharmacol. 2017;27:232–8. doi: 10.1016/j.euroneuro.2017.01.008 28117133 10.1016/j.euroneuro.2017.01.008

[48] Dolder PC, Schmid Y, Mueller F, Borgwardt S, Liechti ME. LSD acutely impairs fear recognition and enhances emotional empathy and sociality. Neuropsychopharmacology. 2016;41:2638–46. doi: 10.1038/npp.2016.82 27249781 10.1038/npp.2016.82 PMC5026740

[49] Dziobek I, Rogers K, Fleck S, Bahnemann M, Heekeren HR, Wolf OT, et al Dissociation of cognitive and emotional empathy in adults with Asperger syndrome using the Multifaceted Empathy Test (MET). J Autism Dev Disord. 2008;38:464–73. doi: 10.1007/s10803-007-0486-x 17990089 10.1007/s10803-007-0486-x

[50] Murphy RO, Ackermann KA, Handgraaf MJJ. Measuring social value orientation Judgment Decision Making. 2011;6:771–81.

[51] Simmler LD, Hysek CM, Liechti ME. Sex differences in the effects of MDMA (ecstasy) on plasma copeptin in healthy subjects. J Clin Endocrinol Metab. 2011;96:2844–50. doi: 10.1210/jc.2011-1143 21715530 10.1210/jc.2011-1143

[52] Nyholt DR. A simple correction for multiple testing for single-nucleotide polymorphisms in linkage disequilibrium with each other. Am J Hum Genet. 2004;74:765–9. doi: 10.1086/383251 14997420 10.1086/383251 PMC1181954

[53] Broadbear JH, Tunstall B, Beringer K. Examining the role of oxytocin in the interoceptive effects of 3,4-methylenedioxymethamphetamine (MDMA, "ecstasy") using a drug discrimination paradigm in the rat. Addict Biol. 2011;16:202–14. doi: 10.1111/j.1369-1600.2010.00267.x 21070509 10.1111/j.1369-1600.2010.00267.x

[54] Thompson MR, Hunt GE, McGregor IS. Neural correlates of MDMA ("Ecstasy")-induced social interaction in rats. Soc Neurosci. 2009;4:60–72. doi: 10.1080/17470910802045042 18633827 10.1080/17470910802045042

[55] Waller R, Corral-Frias NS, Vannucci B, Bogdan R, Knodt AR, Hariri AR, et al An oxytocin receptor polymorphism predicts amygdala reactivity and antisocial behavior in men. Soc Cogn Affect Neurosci. 2016;11:1218–26. doi: 10.1093/scan/nsw042 27036876 10.1093/scan/nsw042 PMC4967804

[56] Bartz JA, Zaki J, Bolger N, Hollander E, Ludwig NN, Kolevzon A, et al Oxytocin selectively improves empathic accuracy. Psychol Sci. 2010;21:1426–8. doi: 10.1177/0956797610383439 20855907 10.1177/0956797610383439 PMC6634294

[57] Apicella CL, Cesarini D, Johannesson M, Dawes CT, Lichtenstein P, Wallace B, et al No association between oxytocin receptor (OXTR) gene polymorphisms and experimentally elicited social preferences. PLoS One. 2010;5:e11153 doi: 10.1371/journal.pone.0011153 20585395 10.1371/journal.pone.0011153 PMC2886839

[58] Heinrichs M, von Dawans B, Domes G. Oxytocin, vasopressin, and human social behavior. Front Neuroendocrinol. 2009;30:548–57. doi: 10.1016/j.yfrne.2009.05.005 19505497 10.1016/j.yfrne.2009.05.005

[59] Seibert J, Hysek CM, Penno CA, Schmid Y, Kratschmar DV, Liechti ME, et al Acute effects of 3,4-methylenedioxymethamphetamine and methylphenidate on circulating steroid levels in healthy subjects. Neuroendocrinology. 2014;100:17–25. doi: 10.1159/000364879 24903002 10.1159/000364879

[60] Parrott A, Lock J, Adnum L, Thome J. MDMA can increase cortisol levels by 800% in dance clubbers. J Psychopharmacol. 2013;27:113–4. doi: 10.1177/0269881112454231 23255436 10.1177/0269881112454231

[61] Chen FS, Barth ME, Johnson SL, Gotlib IH, Johnson SC. Oxytocin receptor (OXTR) polymorphisms and attachment in human infants. Front Psychol. 2011;2:200 doi: 10.3389/fpsyg.2011.00200 21904531 10.3389/fpsyg.2011.00200 PMC3161247

