# Supplementary figures and images for: Oxytocin receptor gene variations and socio-emotional effects of MDMA: A pooled analysis of controlled studies in healthy subjects
Source: PLoS One. 2018 Jun 18;13(6):e0199384. doi: 10.1371/journal.pone.0199384 (PMC6005537; doi:10.1371/journal.pone.0199384)

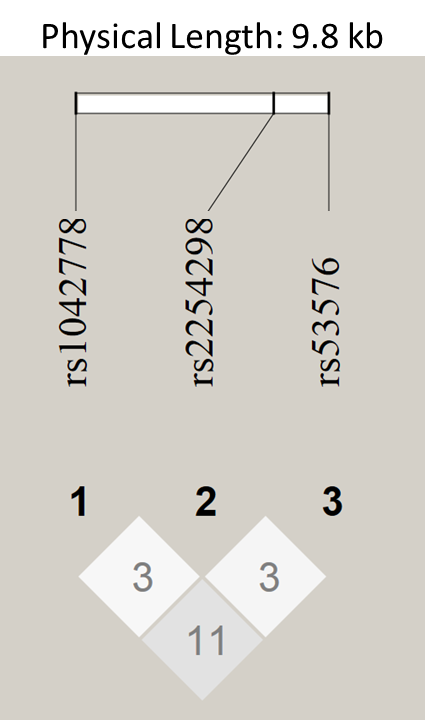

Supplement: S1 Fig — Estimates of the square of the correlation coefficient (r2) were calculated for each pairwise comparison of SNPs based on data from our study cohort. (TIF) [file pone.0199384.s001.tif]
